# Correlating physical activity and quality of life of healthcare workers

**DOI:** 10.1186/s13104-019-4240-1

**Published:** 2019-04-04

**Authors:** Maria Saridi, Theodora Filippopoulou, Georgios Tzitzikos, Pavlos Sarafis, Kyriakos Souliotis, Despoina Karakatsani

**Affiliations:** 1 Nursing Department, General Hospital of Corinth, Corinth, Greece; 2 Faculty of Social and Education Policy, University of Peloponnese, 20131 Corinth, Greece; 3 Sterile Processing Department, General Hospital of Corinth, Corinth, Greece; 4 Renal Unit, Nursing Department, General Hospital of Corinth, 20131 Corinth, Greece; 5 Department of Nursing, School of Health Sciences, Limassol, University of Technology, 3041 Limassol, Cyprus

**Keywords:** Physical activity, Physical exercise, Quality of life, Healthcare worker

## Abstract

**Objective:**

The purpose of the present study was to investigate healthcare workers’ physical exercise levels linked to their quality of life. Healthcare workers’ from all departments of a General hospital participated in the study. The instruments used for data collection regarding quality of life and physical exercise (Short Form Health Survey (SF-36) and International Physical Activity Questionnaire-short form).

**Results:**

Regarding the lack of physical exercise, the participants mainly put the blame on lack of free time (58%, n = 106), work hours (41% n = 75), but also pure negligence (37%, n = 67). The SF-36 scores showed that the existence of health problems can affect in a negative way and aggravate almost every quality of life parameter. Regarding physical activities in the past 7 days prior to the survey, most of them were about housekeeping and household-related chores (42.3%), followed by out-of-the-house errands (13.2%). There were also differences among mental health and postgraduate education level. According to our findings, a major factor that could boost healthcare professionals’ physical activity, is to increase knowledge and raise awareness about the benefits linked to physical activity.

## Introduction

Physical activity (PA) is doubtlessly very important, since it has multiple beneficial effects on physical, mental and spiritual health and wellness. Physical exercise includes any muscular movement or activity that results in calorie loss [1]. Lack of PA is the cause of 6% of deaths worldwide, while a previous study had suggested that the correct percentage was 9% [2].

Despite the well-acknowledged benefits linked to physical exercise, a significant proportion of the global population remains physically inactive. The majority (80%) of adolescents aged 13–15 worldwide regularly fail to reach the targets proposed by the WHO; this proportion is even higher in EU-28, where 83% of teenagers (11–15) are physically inactive [1, 2].

In EU-28, 28.6% of the people do not engage in any kind of physical exercise, while 71.4% are active enough [2, 3]. In Greece, also a typical Mediterranean country, that percentage reached 38.1%, almost ten units above average. On the other hand, Western and Northern Europe had the lowest percentages of physical inactivity, the lowest scores came in from Sweden (12.4%), the Netherlands (14.9%) and Finland (15.9%) [3].

The new PA Strategy for the WHO European Region (2016–2025) seeks to initiate actions on every administrative level that could encourage people to exercise more throughout the life-course, regardless of their gender, age, income, nationality etc. [4]. Healthcare professionals make up a physically and mentally burdened group. Rotating work shifts, demanding tasks, together with family-related issues, make physical exercise hard to be planned and performed. Several studies have highlighted that healthcare professionals do not get enough exercise, but they also indulge in unhealthy food, alcohol abuse and are at a high risk of professional burnout [5, 6].

The aim of the present study was to investigate healthcare workers’ PA levels linked to their Quality of Life (QOL).

## Main text

### Methods

#### Sample

The sample consisted of 180 healthcare professionals working at the General Hospital of Corinth in Greece, from September to November 2016.

#### Instruments

The instruments used for data collection regarding quality of life and physical exercise were:

- The Short Form Health Survey (SF-36) questionnaire [7], measures health status, taking into account physical functioning and exercise, emotional and physical role functioning, mental health in general, social role functioning, body pain and general health.
- The International Physical Activity Questionnaire-short form (self-administered 8-items) [8, 9], which measures days and minutes/day spent on moderate and vigorous activities as well as total walking time for 7 days. It consists of four PA indices: (1) PA during casual walking, (2) moderate-intensity activities, (3) vigorous-intensity activities, (4) total physical activity. The first three indices are calculated by multiplying days per week, minutes per day, and their respective METs (metabolic equivalents) [10]. Total PA is the sum of the above three. The questionnaire also includes demographics data and a question regarding sedentary activities.

#### Ethics

The research protocol was approved by the Ethics Committee of the Hospital (Prot. numb. 284/2/09/2016). The participants signed informed consent forms and anonymity was strictly observed.

#### Statistical analysis

The SPSS 20 was used for the statistical analysis. For the quantitative variables the means, standard deviations (SDs) and medians were used. Absolute and relative frequencies were used for the qualitative variables. Pearson’s Chi squared test was used for comparison of proportions. Student’s *t*-test was used for the comparison of quantitative variables among two groups. For the comparison of quantitative variables among more than two groups, parametric analysis of variance (ANOVA) was the method of choice. The LSD correction was used for the post hoc data analysis. Pearson’s r was used for establishing correlation between two quantitative variables. The correlation is low when r is between 0.1 and 0.3, medium when r is between 0.31 and 0.5, and high if r > 0.5.

### Results

The average age of the sample was 44 years (mean 44.6 ± 6.101), women made up the majority of the sample (84%), and finally most of the participants (> 60%) were of high educational level and married (74%). From a professional point of view, most of the participants belonged to the Nursing Dept. (61%), while regarding their financial situation, 61% (n = 111) thought of it as average, 26% (n = 48) as good, 9% (n = 17) as poor and 4% (n = 7) as very bad.

The participants named *health, family, and mental peace* as the most important factors regarding their quality of life. One out of three of the participants (36%, n = 65) complained about medical conditions, and 26% of them (n = 47) reported long-term medication use.

Regarding the reasons why the participants choose to work out, the main ones were fitness improvement (61.7%, n = 113), stress relief (58.5%, n = 107) and prevention of health problems (55.7%, n = 102). More specifically, most of the participants regarded body weight control as the most important factor (62.8%, n = 115), followed by ameliorating musculoskeletal disorders (40%, n = 73), improving respiratory problems (24%, n = 144), osteoporosis (18.6%, n = 34), blood glucose management (11%, n = 20), and hypertension (8.7%, n = 16).

Regarding the lack of PA, the participants mainly put the blame on lack of free time (58%, n = 106), work hours (41%, n = 75), but also pure negligence (37%, n = 67), and the results showed also low scores on mental health compared to physical health (mean: 59.38 vs 66.49).

Regarding the participants’ gender, females seemed to have statistically significant differences compared to males regarding Vitality (p = 0.049). The participants who chose mental peace as the most important factor for QoL, seemed to score higher physical role functioning too (p = 0.022, t = − 2.302). Those who chose Financial security scored very low on social role functioning (p = 0.016, t = 2.423).

The ANOVA showed a significant difference among the participants’ education level and Physical Functioning (F: 2.710. p = 0.047), Physical Role (F: 3.824, p = 0.011), Mental Health (F: 4.723, p = 0.003) and overall Physical Health (F: 3.156, p = 0.26). The LSD post hoc test showed differences among physical functioning and first-level education (67.0 ± 18,73) and third-level education (82.49 ± 21.32, p = 0.024), as well as among primary education graduates and postgraduates (84.83 ± 17.053, p = 0.017).

There were differences among mental health and postgraduate education level (66.06 ± 14.52), compared to primary education level (48.4 ± 11.99, p = 0.001), and secondary education level (57.82 ± 14.63, p = 0.014); there were also differences among primary and third level graduates (13.98 ± 4.95, p = 0.05). There were also differences among physical health and third-level education (70.26 ± 18.98), secondary education (62.21 ± 18.80. p = 0.013) and primary education (56.25 ± 15.31, p = 0.027), while primary education graduates scored lower than higher-level graduates.

There were also significant differences in the Quality of Life (QoL) taking into account the participants’ financial situation. Those in an average financial situation (50.49 ± 37.50) score lower on Physical Role functioning, compared to those in good financial situation (68.22 ± 34.87, p = 0.007). Those in bad financial situation (37.14 ± 24.640) scored lower in Vitality (F: 3.813, p = 0.11), compared to those in average (53.40 ± 14.97, p = 0.008) and good financial situation respectively (57.55 ± 15.87, p = 0.002).

Almost all of the participants surveyed (90.2%, n = 165) responded positively when asked whether they would like to work out every day. Regarding PA in the past 7 days prior to the survey, most of them were about housekeeping and household-related chores (42.3%), followed by out-of-the-house errands (13.2%). Physical intensity linked with work, commute and housekeeping does not seem to interfere with QoL (Table 1).

**Table 1:** SF-36 variables comparing leisure time activities

SF-36 variables	F	p
PF	7.270	0.001
RE	2.269	0.106
RP	1.888	0.154
VT	11.383	0.000
MT	2.556	0.080
SF	5.152	0.007
BP	4.281	0.015
GH	1.051	0.352
PSC	5.048	0.007
MSC	4.552	0.012

Regarding correlation between level of PA and the participants’ profession, nurses (63.3%) self-reported both vigorous intensity PA and overall PA as well (Table 2). Pearson’s coefficient (r) showed a negative correlation between age and mental health (r: − 0.188, p = 0.011), psychological health (r: − 0.163, p = 0.028), very significant negative correlation with PA (r: − 0.246, p = 0.001) and general health (r: − 0.192, p = 0.009). Also, correlating the total IPAQ scores to QoL categories and age demonstrated a positive correlation between the intensity of physical activity and Vitality (r: 0.157, p: 0.034) as well as social role functioning (r: − 0.171, p = 0.02) (Table 3).

**Table 2:** Total physical activity comparing activity status

Department	Low activity				Medium activity				Intense activity				Total			
	n	Total activity (%)	Department (%)	Participants (%)	n	Total activity (%)	Department (%)	Participants (%)	n	Total activity (%)	Department (%)	Participants (%)	n	Total activity (%)	Department (%)	Participants (%)
Medical	11	13.90	40.70	6.00	13	17.60	48.10	7.10	3	10.00	11.10	1.60	27	14.80	100.00	14.80
Nursing	52	65.80	46.80	28.40	40	54.10	36.00	21.90	19	63.30	17.10	10.40	111	60.70	100.00	60.70
Administrative	14	17.70	37.80	7.70	18	24.30	48.60	9.80	5	16.70	13.50	2.70	37	20.20	100.00	20.20
Technical	2	2.50	25.00	1.10	3	4.10	37.50	1.60	3	10.00	37.50	1.60	8	4.40	100.00	4.40
Total	79	100.00	43.20	43.20	74	100.00	40.40	40.40	30	100.00	16.40	16.40	183	100.00	100.00	100.00

**Table 3:** Pearson’s (r) correlating the total IPAQ scores to QoL categories

	Activity	PSC	MSC	Age	PF	RE	RP	VT	MSC	SF	BP	GH
Total activity	r											
IPAQ	p											
PSC	r	0.004										
	p	0.958										
MSC	r	0.100	*0.602* ^b^									
	p	0.181	*0.000*									
Age	r	− 0.030	− *0.192*^b^	− *0.163*^a^								
	p	0.688	*0.009*	*0.028*								
PF	r	0.055	*0.660* ^b^	*0.271* ^b^	− *0.246*^b^							
	p	0.462	*0.000*	*0.000*	*0.001*							
RE	r	− 0.040	*0.462* ^b^	*0.798* ^b^	− 0.103	*0.165* ^a^						
	p	0.590	*0.000*	*0.000*	0.165	*0.025*						
RP	r	− 0.062	*0.801* ^b^	*0.542* ^b^	− 0.111	*0.290* ^b^	*0.536* ^b^					
	p	0.406	*0.000*	*0.000*	0.135	*0.000*	*0.000*					
VT	r	*0.157* ^a^	*0.462* ^b^	*0.712* ^b^	− 0.099	*0.270* ^b^	*0.284* ^b^	*0.318* ^b^				
	p	*0.034*	*0.000*	*0.000*	0.185	*0.000*	*0.000*	*0.000*				
MSC	r	0.121	*0.304* ^b^	*0.730* ^b^	− *0.188*^a^	0.115	*0.344* ^b^	*0.221* ^b^	*0.708* ^b^			
	p	0.105	*0.000*	*0.000*	*0.011*	0.123	*0.000*	*0.003*	*0.000*			
SF	r	*0.171* ^a^	*0.565* ^b^	*0.799* ^b^	− 0.140	*0.277* ^b^	*0.423* ^b^	*0.427* ^b^	*0.558* ^b^	*0.528* ^b^		
	p	*0.020*	*0.000*	*0.000*	0.059	*0.000*	*0.000*	*0.000*	*0.000*	*0.000*		
BP	r	0.095	*0.805* ^b^	*0.515* ^b^	− 0.114	*0.456* ^b^	*0.317* ^b^	*0.473* ^b^	*0.455* ^b^	*0.333* ^b^	*0.519* ^b^	
	p	0.202	*0.000*	*0.000*	0.125	*0.000*	*0.000*	*0.000*	*0.000*	*0.000*	*0.000*	
GH	r	− 0.066	*0.566* ^b^	*0.316* ^b^	− 0.127	*0.348* ^b^	*0.155* ^a^	*0.235* ^b^	*0.287* ^b^	*0.182* ^a^	*0.388* ^b^	*0.350* ^b^
	p	0.375	*0.000*	*0.000*	0.087	*0.000*	*0.036*	*0.001*	*0.000*	*0.014*	*0.000*	*0.000*

*PF* physical function, *RP* role physical, *BP* bodily pain, *GH* general health, *VT* vitality, *SF* social functioning, *RE* role emotional, *MH* mental health, *PCS* physical component summary, *MCS* mental component summary

^a^Correlation is significant at the 0.05 level (2-tailed)

^b^Correlation is significant at the 0.01 level (2-tailed)

Linear regression gave poor results as far as the prognosis of physical activity. More specifically, the corrected multi-step linear regression model which was used interprets 15.8% of the variance (R square = 0.158) with a dependent variable Total IPAQ, highlighting the economic status, gender and social status of the participants as the main factors (importance 0.40. 0.219 and 0.143 respectively).

### Discussion

Working citizens want a balanced way of life and also the relief through PA with all the resulting benefits. The Randstad Workmonitor survey for the first trimester 2014 showed that employees were more productive and in better shape if they had been exercising systematically. Only a small proportion of them (18%) said that their workplace included a gym or other relevant space [11]. It has been established that activating physical activity and fitness programs for employees could boost productivity and profitability [12, 13]. Some studies have also shown that for every dollar spent for PA programs for the employees, the corporation earned up to $5 [14, 15]. Hospitals are a special workplace that can have a negative impact on the employees physical and mental health. Dealing for many years with medical diseases, rotating shifts, work overload due to understaffing, and physical burden, are reasons that make physical exercise necessary for healthcare providers. The aim of the present study was to investigate the PA of healthcare workers and its correlation to QoL.

According to the results, healthcare workers for the most part had low levels of PA, corresponding to those of the general Greek population [3], and from other Greek healthcare workers as well [16]. There was no significant difference, in our sample, between the intensity of PA (light, moderate, vigorous) and QoL. Nevertheless, the intensity of PA measured in METs seems to have a positive correlation with Vitality and Social Functioning of the participants, which is in agreement with a study from Portugal that showed the positive effect of PA, not so much on QoL itself, but on everyday aspects of life.

For most of the participants physical activity levels were low, which had a correlation to other QoL factors, such as *Physical Functioning, Vitality, Mental Health, Social Functioning, Pain and Physical and Mental Health*. Other relevant studies have also found low levels of physical activity regarding healthcare professionals [17, 18], while doctors seem to have higher levels of PA [19]. Most of our participants (55%) also reported that PA could help them improve their fitness, prevent medical conditions, and reduce stress, a finding supported by other studies too [19, 20]. Especially regarding health issues, the participants preferred body weight control and amelioration of musculoskeletal pain, which is in accordance with other studies [21, 22].

According to the participants, the main reasons preventing them from working out, the lack of free time, the working hours and also negligence were highlighted. Also, working in a hospital is usually a physically taxing job and when combined with demanding household chores, it can reduce one’s willingness to engage in other Physical Activities, which was also supported by other studies [23, 24].

The participants scored lower QoL compared to other studies [25–27]. According to our findings women report lower QoL compared to men and those of a higher educational level reported better QoL, as found by another study [26]. Other studies have shown that female healthcare professionals had had higher levels of PA compared to men, but when they correlated education levels to QoL, their findings were similar to ours. As expected, the existence of medical problems has a negative impact on most of the QoL factors of our participants, which was confirmed by other relevant studies [19, 23].

According to our findings, neither family status nor work assignment are connected to QoL, but Vitality and Physical Role were correlated to the participants’ financial situation, and a major factor that could boost healthcare professionals’ PA, is to increase knowledge and raise awareness about the benefits linked to physical activity [28].

### Conclusions

PA intensity cannot improve the QoL, which is a multidimensional factor. Lack of free time due to the particularities of working in a hospital, is a factor that keeps many employees way from daily physical activities. This fact combined with the willingness of the employees to be more physically active, leads to the result that it would be useful for leisure time management techniques to be developed, both individually and with official policies that could lead to create PA-oriented facilities within the hospitals.

## Limitation of the study

As this is a self reported data, participants may over estimate themselves on PA and QoL status. The method of data collection is open to self-reported and social desirability bias that may affect the result. Further studies on wider and more representative samples of pregnancy women, could yield valid and reliable findings.

## Abbreviations

PA: physical activity
METs: metabolic equivalents
SF-36: short form health survey
QoL: quality of life
